# Metformin protects against insulin resistance induced by high uric acid in cardiomyocytes via AMPK signalling pathways in vitro and in vivo

**DOI:** 10.1111/jcmm.16677

**Published:** 2021-05-30

**Authors:** Zhenyu Jiao, Yingqun Chen, Yang Xie, Yanbing Li, Zhi Li

**Affiliations:** ^1^ Department of Cardiology Beijing Chaoyang Hospital Beijing China; ^2^ Department of Cardiology Second Affiliated Hospital of Shantou University Medical College Shantou, Guangdong China; ^3^ Department of Intensive Care Unit Peking University Shenzhen Hospital Shenzhen China; ^4^ Department of Cardiology Beijing You An Hospital Beijing China

**Keywords:** adenosine 5'‐monophosphate‐activated protein kinase, cardiomyocyte, high uric acid, insulin resistance, metformin

## Abstract

High uric acid (HUA) is associated with insulin resistance (IR) in cardiomyocytes. We investigated whether metformin protects against HUA‐induced IR in cardiomyocytes. We exposed primary cardiomyocytes to HUA, and cellular glucose uptake was quantified by measuring the uptake of 2‐NBDG, a fluorescent glucose analog. Western blot was used to examine the levels of signalling protein. Membrane of glucose transporter type 4 (GLUT4) was analysed by immunofluorescence. We monitored the impact of metformin on HUA‐induced IR and in myocardial tissue of an acute hyperuricaemia mouse model established by potassium oxonate treatment. Treatment with metformin protected against HUA‐reduced glucose uptake induced by insulin in cardiomyocytes. HUA directly inhibited the phosphorylation of Akt and the translocation of GLUT4 induced by insulin, which was blocked by metformin. Metformin promoted phosphorylation of AMP‐activated protein kinase (AMPK) and restored the insulin‐stimulated glucose uptake in HUA‐induced IR cardiomyocytes. As a result of these effects, in a mouse model of acute hyperuricaemia, metformin improved insulin tolerance and glucose tolerance, accompanied by increased AMPK phosphorylation, Akt phosphorylation and translocation of GLUT4 in myocardial tissues. As expected, AICAR, another AMPK activator, had similar effects to metformin, demonstrating the important role of AMPK activation in protecting against IR induced by HUA in cardiomyocytes. Metformin protects against IR induced by HUA in cardiomyocytes and improves insulin tolerance and glucose tolerance in an acute hyperuricaemic mouse model, along with the activation of AMPK. Consequently, metformin may be an important potential new treatment strategy for hyperuricaemia‐related cardiovascular disease.

## 1 INTRODUCTION

Insulin resistance usually refers to a clinicopathological condition characterized by an impaired ability of insulin to stimulate glucose uptake and by glucose intolerance.^1^ Moreover, insulin resistance is closely related to several cardiovascular diseases, such as myocardial infarction, hypertension and heart failure.^2^, ^3^, ^4^ Hyperuricaemia occurs together with abnormal glucose metabolism and also insulin resistance; it participates in the pathological process of cardiovascular diseases and thereby induces a variety of complications that may affect the quality of life of patients. Clinical follow‐up studies have demonstrated that elevated serum uric acid is a strong independent risk factor for insulin resistance,^5^ and ours and others’ previous studies have revealed that high uric acid (HUA) evokes insulin resistance in cardiomyocytes, adipocytes, muscle and liver.^6^, ^7^, ^8^, ^9^ However, the therapeutic strategies of hyperuricaemia are limited and diversified treatment requires further research.

Adenosine 5'‐monophosphate‐activated protein kinase (AMPK) is an essential metabolic regulator for energy sensing and has been well documented.^10^ AMPK is expressed specifically in various tissues and cells, including cardiomyocytes,^11^ and plays a crucial role in regulating energy metabolism and energy homeostasis under stress conditions. AMPK can be activated by various conditions. Activation of AMPK phosphorylation regulates a series of metabolic steps by inducing the ATP generation system (such as glycolysis and fatty acid oxidation) and inhibiting the ATP consumption system (such as the synthesis of fatty acids). Our previous data demonstrated that AMPK is activated by HUA via oxidative stress in pancreatic β cells.^12^ In our previous study, HUA induced oxidative stress and played a critical role in the development of insulin resistance and its potential role in the pathogenesis of metabolic stress in cardiomyocytes.^6^ Moreover, phosphorylated Akt‐mediated insulin signal transduction and translocation of glucose transporter type 4 (GLUT4) were suppressed by HUA and were related to insulin resistance and impaired glucose uptake in cardiomyocytes.^6^

Metformin, well known as an AMPK activator and as an antidiabetic medicine, is widely used and has various pharmacologic effects, including promoting glucose uptake and utilization, enhancing insulin sensitivity and preventing hyperlipidaemia.^13^ Major advances in the scientific understanding of metformin action have focused on the discovery that metformin leads to the activation of phospho‐AMPK. This activation of phospho‐AMPK appears to be more associated with changes in phosphocreatine levels and the AMP/ATP ratio. The AMPK system acts as an energy‐sparing sensor, and therefore, AMPK is a good mediator of metformin activity on heightened glucose uptake and the improved cellular status of energy that follows glucose uptake.

In our previous study, we found that HUA induced insulin resistance and suppressed glucose uptake in cardiomyocytes in vitro and in vivo.^6^ In the present study, we assessed whether there are positive effects of metformin's performance on glucose uptake inhibited by HUA in cardiomyocytes and whether treatment with metformin can either circumvent or reverse insulin resistance induced by HUA. At the cellular level, insulin resistance can be induced by prolonged incubation with HUA in cardiomyocytes. The impacts of HUA may be related to reductions in insulin signalling events at the level of phosphatidylinositol 3‐kinase activation of Akt.

At the molecular level, insulin resistance is characterized by an impaired insulin‐activated insulin receptor substrate/phosphoinositide 3‐kinase/Akt (IRS‐PI3K‐Akt) pathway, the major player in the metabolic action of insulin, which leads to suppressed insulin‐induced glucose uptake in insulin‐sensitive organs, including the heart.^14^, ^15^ In our previous study, we found that HUA inhibited insulin signalling and suppressed glucose uptake, and Akt mediates insulin‐stimulated glucose uptake in cardiomyocytes in vitro and in vivo.^6^ In the present study, we assessed whether there are positive effects of metformin's performance on glucose uptake inhibited by HUA in cardiomyocytes and whether treatment with metformin can either circumvent or reverse insulin resistance induced by HUA. At the cellular level, insulin resistance can be induced by prolonged incubation with HUA in cardiomyocytes. The impacts of HUA may be related to reductions in insulin signalling events at the level of phosphatidylinositol 3‐kinase activation of Akt.

Previous studies have demonstrated that metformin can protect the heart by inhibiting apoptosis, autophagy, inflammation and other pathways via AMPK.^9^, ^11^, ^16^ However, whether metformin protects against insulin resistance induced by HUA in cardiomyocytes remains unknown. Therefore, we determined whether metformin activation of the AMPK‐dependent pathway can protect against insulin resistance induced by HUA in cardiomyocytes.

## 2 METHODS AND MATERIALS

### 2.1 Reagents

Uric acid (15 mg/dL, final concentration), metformin, insulin (100 nM, final concentration) and compound C (20 μM, final concentration) were purchased from Sigma. N‐(7‐Nitrobenz‐2‐oxa‐1,3‐diazol‐4‐yl)amino]‐2‐deoxy‐d‐glucose (2‐NBDG, 100 μM, final concentration), Alexa Fluor®–conjugated secondary antibodies, anti‐IRS1 and anti‐phospho‐IRS1 (Ser307) antibodies were purchased from Invitrogen. 5‐Amino‐4‐imidazole‐1‐β‐D‐carboxamide ribofuranoside (AICAR, 500 μM, final concentration) was purchased from MedChemExpress (MCE LLC, USA). A rabbit anti‐GAPDH and anti‐GLUT4 antibody were purchased from Abcam (Abcam, USA). Anti‐Akt (Ser473) and anti‐phospho‐Akt (Ser473) antibodies were purchased from Bioworld (St. Louis Park, USA). Anti‐AMPK (Thr172) and anti‐phospho‐AMPK (Thr172) antibodies were purchased from Cell Signaling Technology (CST, Beverly). Cat no for antibodies: anti‐phospho‐IRS1 (Ser307) (44‐813G); anti‐phospho‐AMPK (Thr172) (50081); anti‐phospho‐Akt (Ser473) (APG01691G); anti‐GLUT4 (ab33780).

### 2.2 Cell culture

Cellular studies were carried out on primary cardiomyocytes isolated from C57BL/6 neonatal mice according to a previous protocol.^17^ Cardiomyocytes were incubated with Dulbecco's modified Eagle medium (DMEM) or low‐glucose minimum essential medium eagle (MEM) containing L‐glutamine (2 mM), streptomycin (100 mg/mL), penicillin G (100 U/mL) and 10% foetal bovine serum. For all experiments, the cardiomyocytes were seeded into 6‐well plates at a density of 2.0 × 10^5^ cells/mL. Primary cardiomyocytes were incubated in a 37℃ incubator with 95% air and 5% CO_2_. Cardiomyocytes were cultured in serum‐free media for 24 hours and then incubated in the presence of 15 mg/dL HUA for 24 hours. Cardiomyocytes were pre‐treated with either metformin (0 to 20 μmol/L) or AICAR, an AMPK activator (500 μmol/L), for 60 minutes before the addition of HUA. Other cells were pre‐incubated with compound C for 60 minutes before the addition of either metformin or AICAR. Then, 2‐NBDG uptake was analysed in cardiomyocytes.

### 2.3 Uptake of 2‐NBDG glucose was measured by fluorescence microscopy and flow cytometry

A fluorescent glucose analog, 2‐NBDG, glucose uptake, was measured by fluorescence microscopy and flow cytometry in cardiomyocytes to assess glucose uptake.

Briefly, for fluorescence microscopy protocol,^6^ the cells were incubated in foetal bovine serum–free medium for 24 hours. The culture medium was replaced by Krebs‐Ringer bicarbonate buffer containing insulin (100 nM) and 2‐NBDG (100 μM) at 37℃ for 30 minutes and analysed at excitation and emission wavelengths of 488 and 525 nm, respectively.

For flow cytometry, the cells were fed low‐glucose DMEM without foetal bovine serum for 24 hours. The culture medium was then replaced with Krebs‐Ringer bicarbonate (KRB) buffer containing 2‐NBDG (100 μM) and insulin (100 nM) for 30 minutes at 37℃ in a CO_2_ incubator. For flow cytometry–based 2‐NBDG glucose uptake assays, after treatment, 2‐NBDG was washed out of the culture medium, and uptake of 2‐NBDG was assessed by flow cytometry with a fluorometer at an excitation wavelength of 488 nm and an emission wavelength of 525 nm. For each sample, 20 000 cells in each well were obtained in the FSC x SSC plots.

### 2.4 GLUT4 translocation assay

Plasma membrane expression of GLUT4 was determined in cardiomyocytes under non‐permeabilizing conditions. After differentiation, cardiomyocytes were further cultured in a serum‐free medium for 4 hours and stimulated for 30 minutes with 100 nM insulin. Where indicated, cells were incubated with HUA, metformin, ICAR and/or compound C prior to stimulation with insulin. Cells fixed in paraformaldehyde were sequentially incubated with anti‐GLUT4 antibody and Alexa Fluor®–conjugated secondary antibodies. After decoration with the antibodies, observed with a Leica confocal microscope, and representative images are shown. Quantification was blinded using ImageJ (National Institutes of Health) for image processing.

### 2.5 Transfection with siRNA for AMPK

We used small interfering RNA (siRNA) for AMPK in order to knock down the protein of endogenous AMPK. The siRNA for the AMPK (siRNA‐AMPK) was purchased from Santa Cruz Biotechnology (Shanghai). For siRNA‐AMPK experiments, cardiomyocytes were grown on gelatine pre‐coated 6‐ or 24‐well microplates for 24 hours. Transfection with siRNA‐AMPK was performed using Lipofectamine 2000. To knock down AMPK expression, the cells were grown for 24 hours after transfection. Later, cardiomyocytes were starved of FBS overnight, and glucose uptake and GLUT4 detection assays were performed. AMPK down‐regulation was confirmed by performing Western blot analysis.

### 2.6 Animal experiments

The protocol was unanimously affirmed by the Shantou University Animal Experiments Ethics Committee (Permit Number: SUMC2017‐049). All methods were performed in accordance with the relevant guidelines. C57BL/6J male mice (8 weeks old) were obtained from Vital River Laboratories Animal, Ltd. (Beijing, China) in this study. All animals were housed in the Laboratory Animal Center of Shantou University Medical College. The animals were housed in cages under a 12‐hour light‐dark cycle and fed water and a normal food diet. Cardiac myocytes and ventricle cardiac tissue were obtained as previously reported.^18^

### 2.7 Experimental acute hyperuricaemia mouse model establishment and metformin treatment

Ten‐week‐old male mice were randomly divided into three groups (n = 8 each) for treatment: the control, HU (hyperuricaemia) and HU‐Met (metformin) groups. For the HU group, the uricase inhibitor potassium oxonate was used to induce hyperuricaemia in mice according to previous report.^17^ Following an 18‐hour overnight fast, the mice were injected intraperitoneally (i.p.) with 300 mg/kg potassium oxonate and intragastrically with 500 mg/kg hypoxanthine to create the model of acute hyperuricaemia for 1‐2 hours.^19^ The mice in the HU‐Met group were administered 250 mg/kg/day metformin in drinking water for 14 consecutive days.^20^ The quantity of medicine was based on bodyweight levels before each dose. The level of serum uric acid was determined by the phosphotungstic acid method at different times.^19^ Insulin tolerance tests (ITTs) and glucose tolerance tests (GTTs) were performed as described previously.^8^ For the control group and HU group, the mice were killed by CO_2_ inhalation 10 minutes after injection with 2 U/kg insulin. Cardiac muscle tissue was excised and stored in liquid nitrogen immediately.

### 2.8 Western blot

The cultured cardiomyocytes and heart tissues were washed twice with cold PBS, harvested, lysed in ice‐cold lysis buffer (pH = 7.4, 100 mM KCl, 10 mM HEPES, 1.5 mM MgCl), sonicated, homogenized and then subjected to protein extraction with RIRP buffer with a phosphatase inhibitor cocktail and a protease inhibitor cocktail. After centrifugation, a Pierce BCA protein assay kit was used to determine the protein concentration. Equal concentrations of soluble lysate protein (50 μg) were added to a 12% SDS‐PAGE Bis‐Tris gel, and the protein was transferred to immunoblot polyvinylidene fluoride (PVDF) membranes (Bio‐Rad). Membranes were incubated and blocked overnight at 4℃ with antibodies against p‐AMPK/AMPK, p‐Akt/Akt and GAPDH. Then, the membranes were incubated with anti‐rabbit horseradish peroxidase–conjugated secondary antibodies (1:10 000 dilution). The immunoreactive bands were analysed with an enhanced chemiluminescence substrate kit (Biological Industries, BI).

### 2.9 Statistical analysis

Data are presented as the means ± SD. Significant differences between the means were analysed by Student's *t* test. Significant differences were evaluated using one‐way analyses of variance (ANOVA), and *P* values less than .05 were considered to be statistically significant.

## 3 RESULTS

### 3.1 Metformin stimulated the phosphorylation of AMPK and attenuated insulin resistance induced by HUA in cardiomyocytes

**FIGURE 1:**
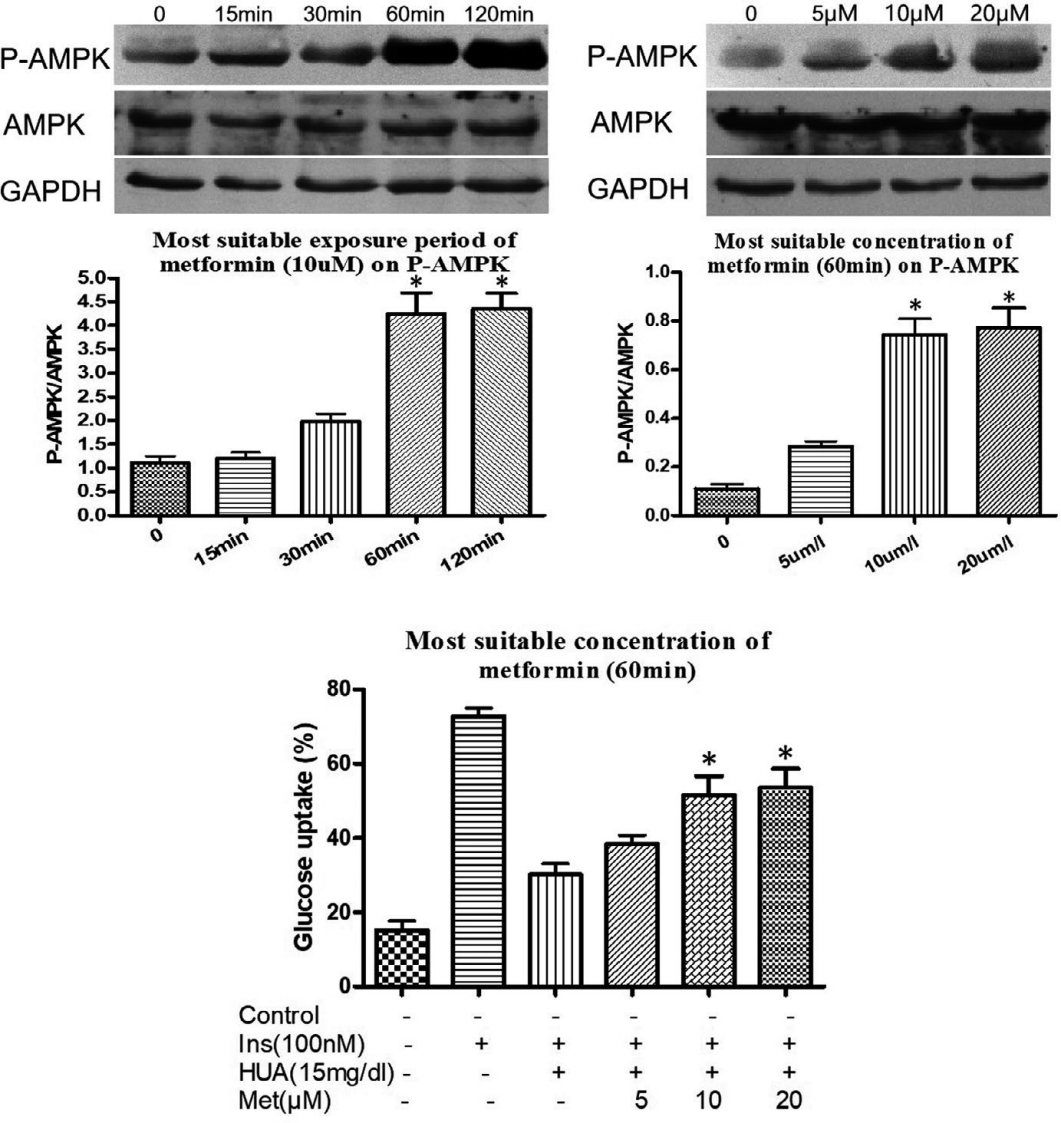
Effect of metformin on AMPK phosphorylation (A‐B) and HUA‐inhibited glucose uptake induced by insulin in primary cardiomyocytes (C). (A‐B) Western blot analysis of phosphorylated and total AMPK levels. (C) Pre‐treated with metformin (10 μmol/L) for 60 minutes protected against HUA (15mg/dL for 24 hours) inhibited 2‐NBDG uptake induced by insulin in primary cardiomyocytes, as shown by flow cytometry analysis. (A) *P < .05 vs. 0, 15 and 30 min. (B) *P < .05 vs. 0 and 5uM. **(C)** *P < .05 vs. Ins+HUA. Data are mean ± SD from 4 separate experiments. Ins: insulin. HUA: high uric acid. Met: metformin. 2‐NBDG: 2‐[N‐(7‐Nitrobenz‐2‐oxa‐1,3‐diazol‐4‐yl)amino]‐2‐deoxy‐d‐glucose

We first wanted to determine the most suitable exposure period and concentration of metformin for the activation of phospho‐AMPK in cardiomyocytes. Treatment with metformin (using the most suitable concentration and exposure period of 10 μM for 60 minutes) stimulated AMPK phosphorylation in cardiomyocytes in a dose‐ and time‐dependent manner (Figure 1A,B). Moreover, 2‐NBDG glucose uptake was decreased significantly in the presence of HUA (15 mg/dL) for 24 hours in cardiomyocytes, as shown by the flow cytometry assay. However, metformin could significantly ameliorate the HUA‐reduced glucose uptake in a dose‐ and time‐dependent manner in cardiomyocytes (Figure 1C). These results demonstrated that HUA inhibited 2‐NBDG glucose uptake induced by insulin and caused insulin resistance in primary cardiomyocytes, but this change was attenuated by treatment with metformin.

### 3.2 Metformin attenuated insulin resistance induced by HUA via AMPK signalling pathways in cardiomyocytes

**FIGURE 2:**
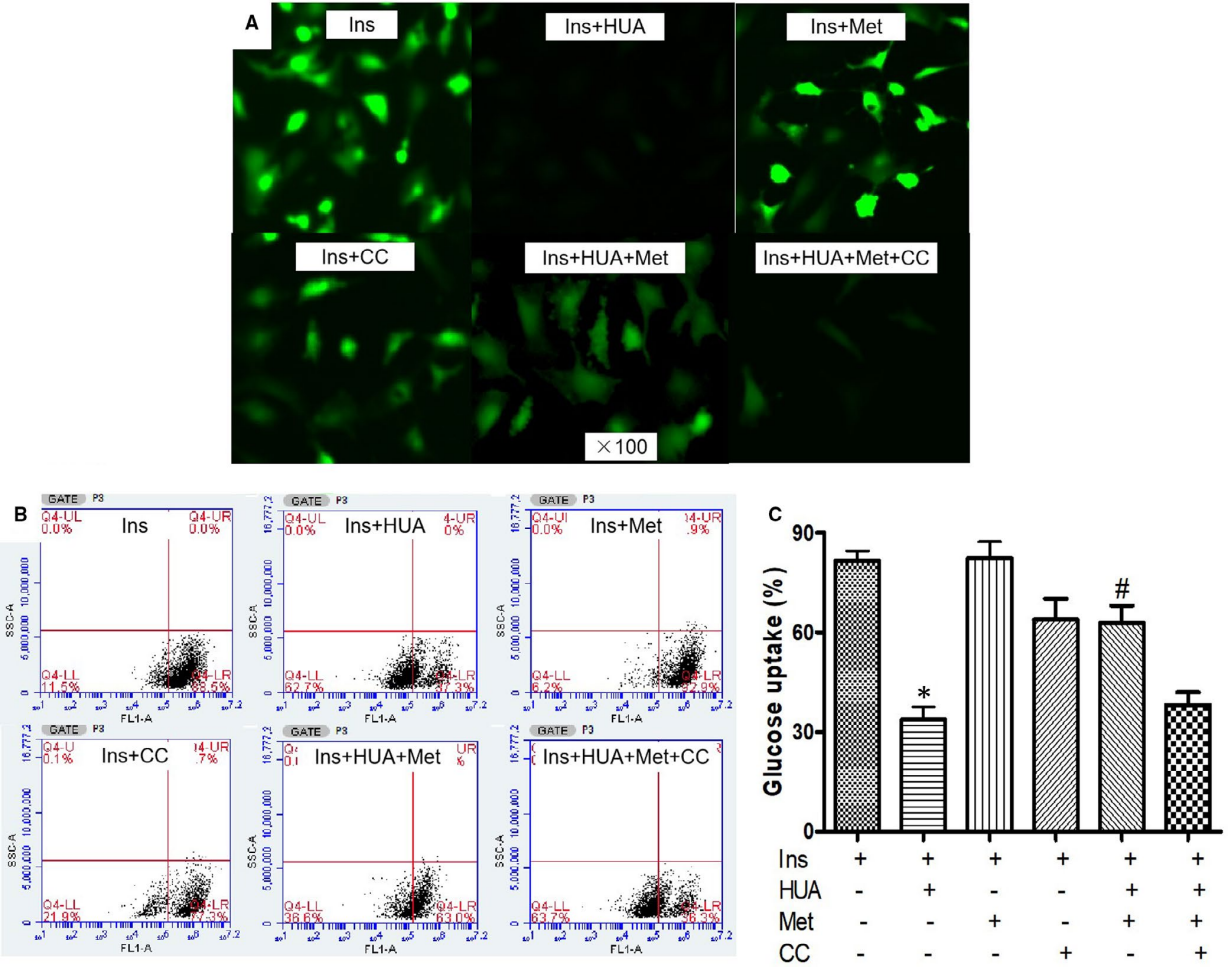
Metformin protected against HUA inhibited 2‐NBDG uptake induced by insulin via AMPK signal pathways in primary cardiomyocytes. (A) 2‐NBDG uptake assay detected by fluorescence microscopy. (B‐C) 2‐NBDG uptake assay detected by flow cytometry. *P < .05 vs. Ins and Ins+HUA+Met, #*P* < .01 vs. Ins+HUA+Met+CC. Data are mean ± SD from 4 separate experiments. CC: compound C, an AMPK inhibitor

To investigate whether metformin attenuates HUA‐induced insulin resistance via AMPK signalling pathways in cardiomyocytes, cardiomyocytes were pre‐treated with metformin (10 μM) for 60 minutes and/or compound C (20 μM), a specific AMPK inhibitor, for 6 hours before exposure to HUA. Under these conditions, the effect of metformin on 2‐NBDG glucose uptake was blunted by co‐treatment with compound C (Figure 2A,C).

**FIGURE 3:**
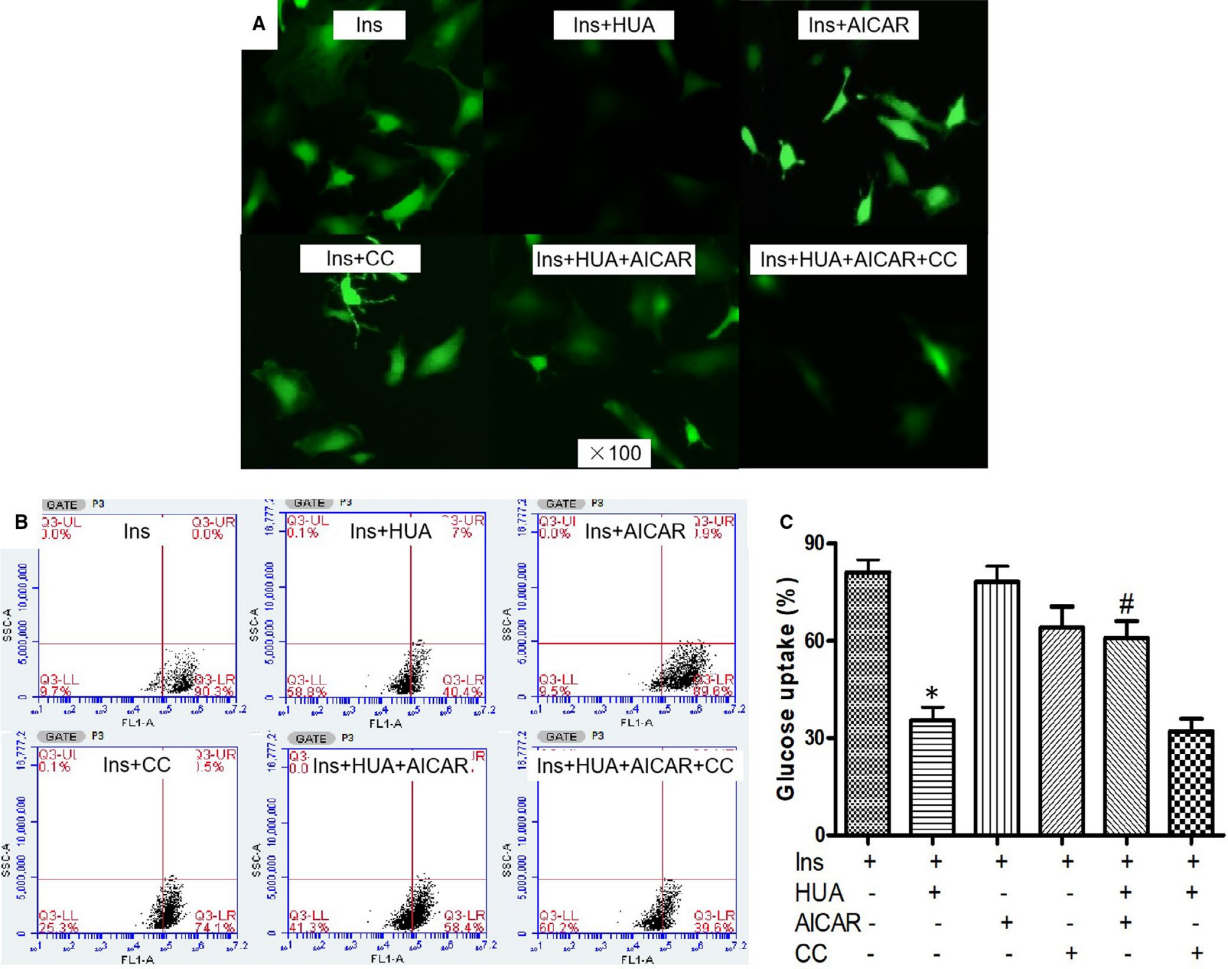
5‐amino‐4‐imidazole‐1‐β‐D‐carboxamide ribofuranoside (AICAR, another AMPK activator) protected against HUA inhibited 2‐NBDG uptake induced by insulin via AMPK signal pathways in primary cardiomyocytes. (A) 2‐NBDG uptake assay detected by fluorescence microscopy. (B‐C) 2‐NBDG uptake assay detected by flow cytometry. *P < .05 vs. Ins and Ins+HUA+AICAR, #*P* <.01 vs. Ins+HUA+AICAR+CC. Data are mean ± SD from 4 separate experiments

To confirm the role of AMPK signalling pathways in metformin attenuation of HUA‐induced insulin resistance in cardiomyocytes, cells were pre‐treated with another AMPK activator, AICAR (500 μM), for 60 minutes before exposure to HUA. We found that AICAR had an effect similar to metformin on glucose (2‐NBDG) uptake after exposure to HUA (Figure 3A,C). These findings demonstrated that activation of AMPK signalling pathways protected against insulin resistance induced by HUA in cardiomyocytes.

### 3.3 Metformin increased phospho‐AMPK, phospho‐Akt and translocation of membrane GLUT4

**FIGURE 4:**
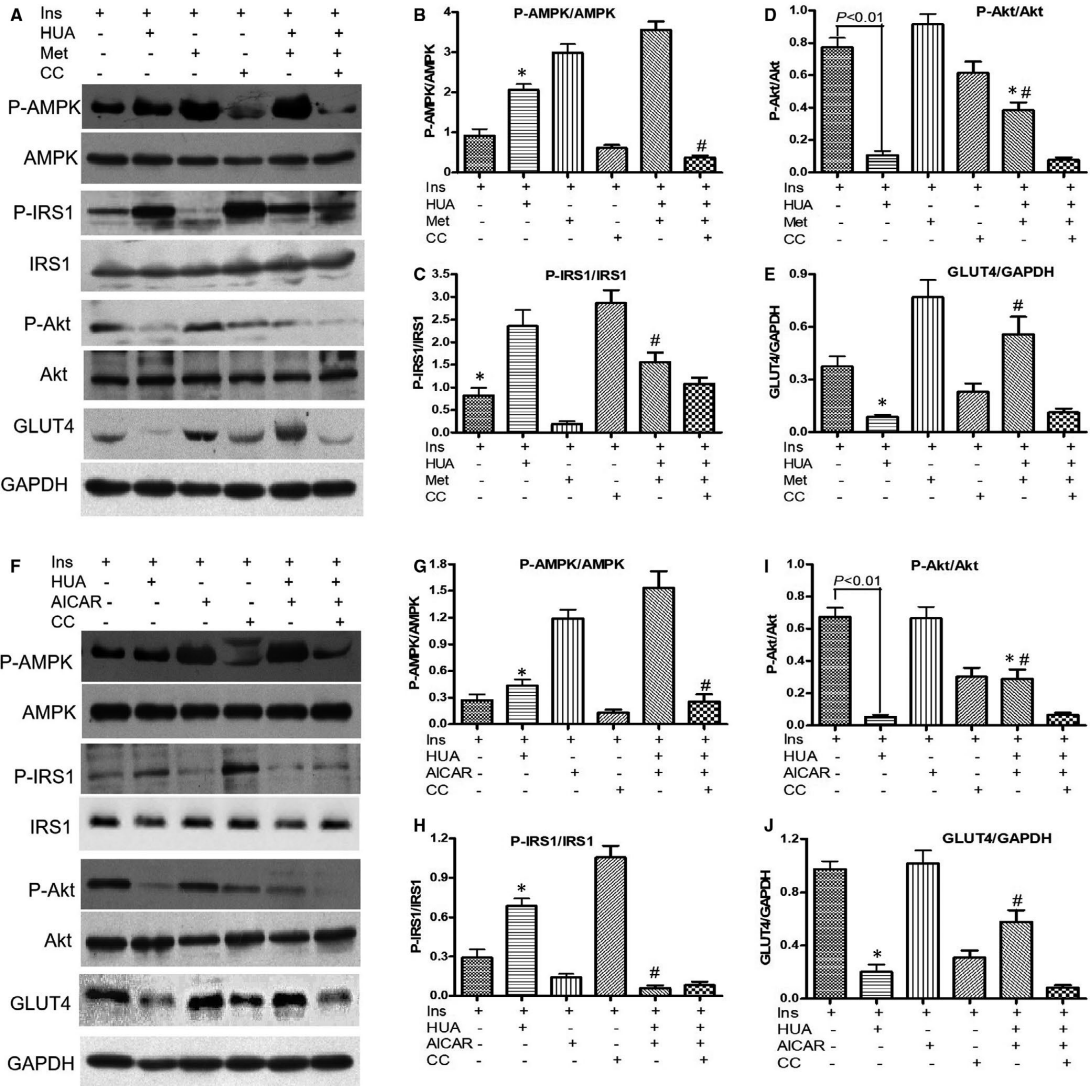
(A‐E) Effect of metformin on HUA‐induced/inhibited of phospho‐AMPK, phospho‐IRS1, phospho‐Akt and translocation of membrane GLUT4 in primary cardiomyocytes. (B) *P < .05 vs. Ins and Ins+HUA+Met; #*P* < .01 vs. Ins+HUA+Met. (C) *P < .05 vs. Ins+HUA and Ins+CC; #*P* < .01 vs. Ins+HUA+Met. (D) *P < .05 vs. Ins+HUA, Ins+Met and Ins+HUA+Met+CC. (E) *P < .05 vs. Ins and Ins+HUA+Met; #*P* < .01 vs. Ins+HUA. (F‐J) Effect of AICAR on HUA‐induced/inhibited of phospho‐AMPK, phospho‐IRS1, phospho‐Akt and translocation of membrane GLUT4 in primary cardiomyocytes. G, *P < .05 vs. Ins and Ins+HUA+Met; #*P* < .01 vs. Ins+HUA+Met. (H) *P < .05 vs. Ins+HUA and Ins+CC. (I) *P < .05 vs. Ins+HUA, Ins+Met and Ins+HUA+Met+CC. (J) *P < .05 vs. Ins and Ins+HUA+Met; #*P* < .01 vs. Ins+HUA

AMPK is a well‐known master energy sensing regulator. Furthermore, the Akt family of Thr/Ser protein kinases is of critical importance with regard to cardiac metabolism and growth in insulin signal transduction. Consequently, to further inspect the molecular mechanism of the AMPK and Akt signalling pathways, we first examined whether metformin activated AMPK in cardiomyocytes. In cardiomyocytes, metformin enhanced the phosphorylation of AMPK, and this phosphorylation was blocked by compound C, a specific AMPK inhibitor (Figure 4,B).

**FIGURE 5:**
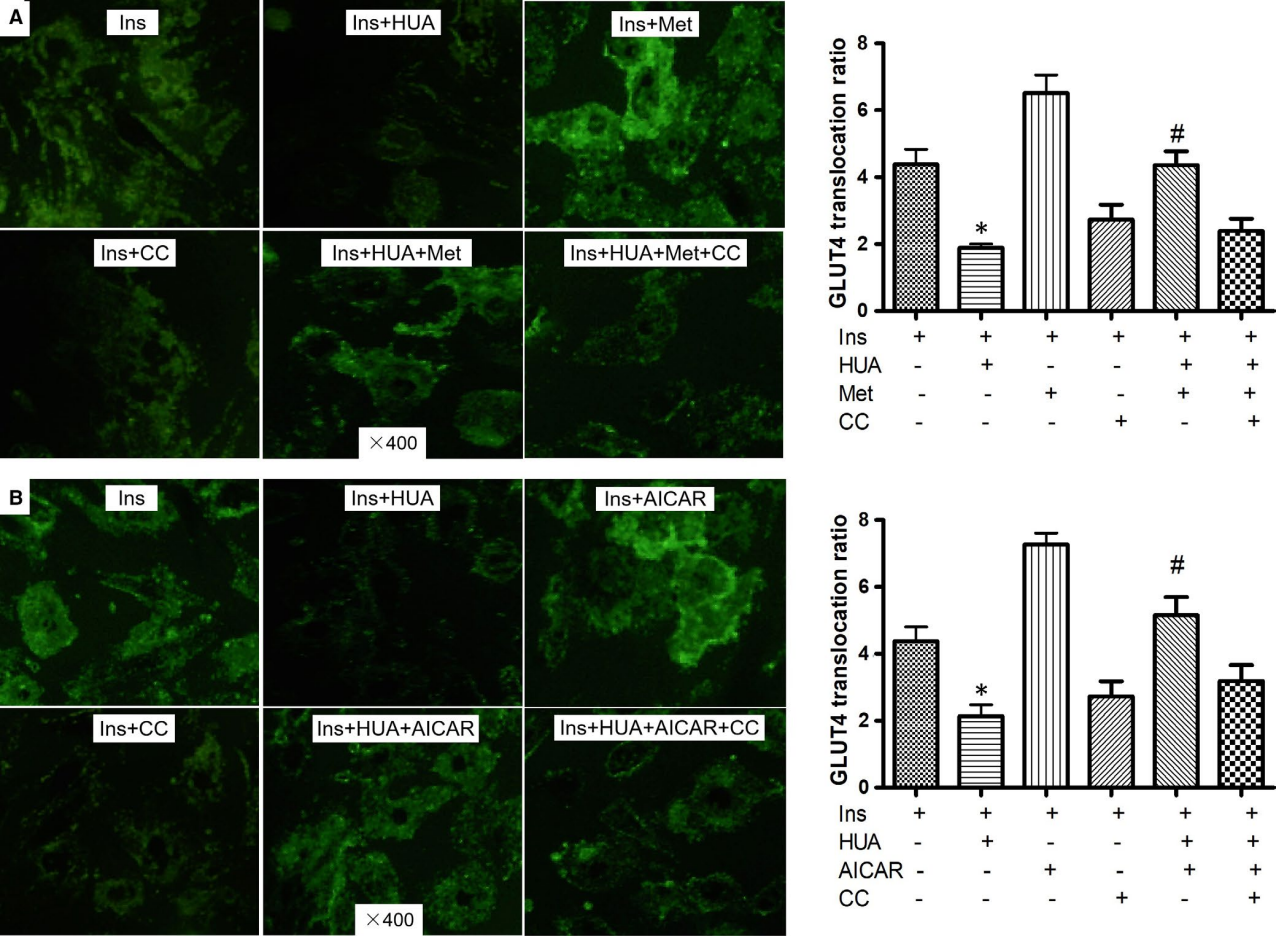
Effect of metformin (A) or AICAR (B) on HUA‐induced/inhibited redistribution of GLUT4 protein in primary Cardiomyocytes. GLUT4 expression (green fluorescent protein, GFP) on plasma membranes (PM) was determined using anti‐GLUT4 antibody and Alexa Fluor®–conjugated secondary antibody under impermeable staining conditions. The images were taken using a Leica confocal microscope. Quantification was blinded using ImageJ (National Institutes of Health) for image processing. PM localization was determined to score 20 representative cells per condition for the appearance of a PM ring of GLUT4. The ratio of GLUT4 translocation was calculated by fluorescence on PM/GFP fluorescence in whole cardiomyocytes. (A‐B) *P < .05 vs. Ins; #*P* < .05 vs. Ins+HUA

To test whether metformin also activated the expression of phospho‐Akt and translocation of membrane GLUT4 in cardiomyocytes, we determined phospho‐Akt expression and translocation of membrane GLUT4 by performing Western blot analyses in cardiomyocytes. We demonstrated that the insulin‐induced phosphorylation level of Akt (Figure 4A, Figure 4D) and translocation of membrane GLUT4 (Figure 4A, Figure 4E, Figure 5A) were significantly inhibited by HUA in cardiomyocytes. As compared with other phosphorylated forms of IRS1 (Ser307), which activate insulin signalling, phospho‐IRS1 (Ser307) inhibits insulin signalling. In this study, HUA exposure increased phospho‐IRS1 (Ser307) level in cardiomyocytes and metformin attenuated significantly (Figure 4A, Figure 4C). Interestingly, AICAR had an effect similar to metformin on phospho‐IRS1 (Ser307) (Figure 4F, Figure 4H). These results indicated that metformin protects against insulin resistance induced by HUA in cardiomyocytes via AMPK/IRS1 signalling pathways.

Furthermore, we investigated the role of metformin in HUA‐mediated inhibition of Akt activation and GLUT4 translocation. Metformin did not influence the insulin‐induced activation of Akt phosphorylation (Figure 4A, Figure 4D) or translocation of GLUT4 (Figure 4A, Figure 4E, Figure 5A). However, insulin‐induced activation of Akt phosphorylation (Figure 4A, Figure 4D) and translocation of membrane GLUT4 (Figure 4D, Figure 5A) were depressed by HUA, which was attenuated significantly by metformin (Figure 4A, Figure 4E, Figure 5A). Nevertheless, the effect of HUA on Akt phosphorylation and translocation of GLUT4 was activated by metformin, which was significantly offset by compound C, an AMPK inhibitor (Figure 4A, Figure 4C,E, Figure 5A). As expected, we found that AICAR had an effect similar to metformin on phospho‐AMPK (Figure 4F, Figure 4G), phospho‐Akt (Figure 4F, Figure 4I) and translocation of membrane GLUT4 (Figure 4F, Figure 4J, Figure 5B) after exposure to HUA. These results demonstrated a profound effect of metformin on downstream targets of the insulin signalling pathway via AMPK.

### 3.4 siRNA‐AMPK abolished the increase in translocation of membrane GLUT4 and glucose uptake that was induced by using metformin and AICAR

**FIGURE 6:**
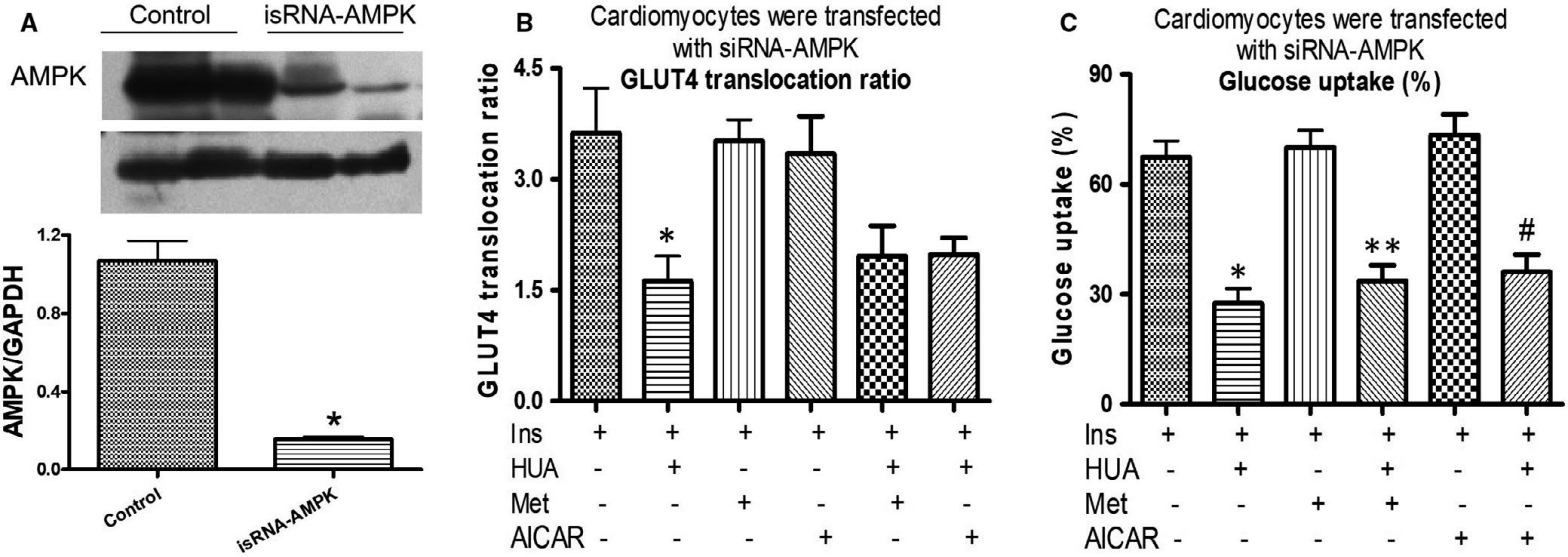
(A) Cardiomyocytes were transfected with siRNA‐AMPK, and AMPK down‐regulation was confirmed by performing Western blot analysis. GAPDH was used as the protein loading control. *P < .01 vs. Con. (B) Effect of metformin or AICAR on HUA‐induced/inhibited redistribution of GLUT4 protein in cardiomyocytes transfected with siRNA for AMPK. *P < .05 vs. Ins. (C) Effect of metformin or AICAR on HUA‐inhibited glucose uptake induced by insulin in cardiomyocytes transfected with siRNA for AMPK. *P < .05 vs. Ins; **P < .05 vs. Ins+Met; #*P* < .05 vs. Ins+AICAR

In order to better understand how AMPK activation protected against insulin resistance induced by HUA in cardiomyocytes, we used siRNA for AMPK to knock down the protein of endogenous AMPK. The knock‐down AMPK expression efficiency was checked by performing Western blotting, and the Western blot results revealed that si‐AMPK transfection significantly reduced the expression of AMPK compared with control (Figure 6A). Moreover, siRNA‐AMPK abolished the increase in translocation of membrane GLUT4 (Figure 6B) and glucose uptake (Figure 6C) that was induced by using metformin and AICAR. These findings further demonstrated that metformin protects against insulin resistance induced by HUA in cardiomyocytes via AMPK signalling pathways.

### 3.5 Effect of metformin in a hyperuricaemia mice model

#### 3.5.1 Effect of metformin on insulin resistance induced by hyperuricaemia in a mouse model

**FIGURE 7:**
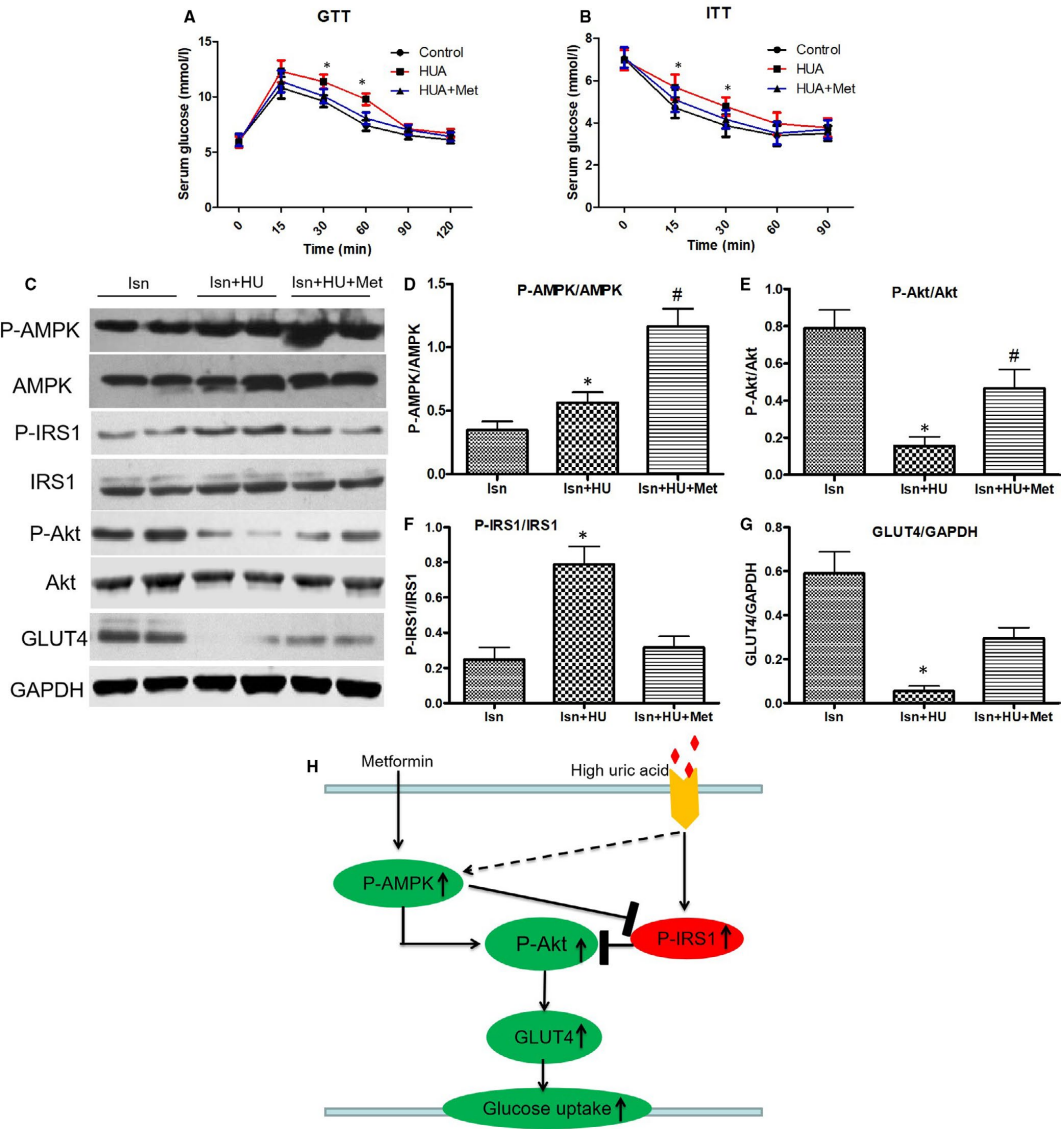
**(**A‐B) Glucose tolerance test (GTT) (A) and insulin tolerance test (ITT) (B) in an acute hyperuricaemic mice model. *P < .05 vs. Con and HU+Met. (C‐G) Western blot analysis of phospho‐AMPK, phospho‐IRS1, phospho‐Akt and GLUT level in cardiac tissues. (D) *P < .05 vs. Ins+HU. (E) *P < .05 vs. Ins and Ins+HU+Met. (F) *P < .05 vs. Ins and Ins+HU+Met. (G) *P < .05 vs. Ins and Ins+HU+Met. Data are mean ± SD from 4 separate experiments. (H) Schematic representation of how metformin ameliorates HUA‐induced insulin resistance in cardiomyocytes. After treatment with HUA, which activates phosphorylation of IRS1 (Ser307). This activity impairs AKT (Ser473) phosphorylation, for insulin resistance. After treatment with metformin, the phosphorylation of AMPK is increased. At the same time, this activity reverses the inhibition of HUA‐induced AKT phosphorylation, which ameliorates HUA‐induced insulin resistance. HU: hyperuricaemia. Met: metformin

As expected, the levels of serum uric acid in the acute hyperuricaemia mouse model were significantly higher than those before hyperuricaemia induction (115.69 ± 18.32 vs 39.71 ± 4.43 mg/L), which was consistent with primary hyperuricaemia patients.^21^ Our acute hyperuricaemia mouse model also indicated an impaired glucose tolerance test (Figure 5A) and insulin tolerance test (Figure 5B) at 15 and 30 minutes after insulin or glucose injection with insulin resistance. Treatment with metformin significantly decreased the level of serum glucose in the glucose tolerance test and insulin tolerance test compared with the hyperuricaemia mouse model (Figure 7A,B). Thus, these findings demonstrate that metformin protects against hyperuricaemia‐induced insulin resistance in a mouse model.

#### 3.5.2 Effect of metformin on AMPK, IRS1 phosphorylation, Akt phosphorylation and translocation of GLUT4 in cardiac tissues from the acute hyperuricaemic mouse model

To examine the molecular mechanisms by which metformin protects against hyperuricaemia‐induced insulin resistance in cardiomyocytes in an acute hyperuricaemic mouse model, we investigated the effect of metformin on AMPK, IRS1 phosphorylation, Akt phosphorylation and GLUT4 translocation in cardiac tissues from the acute hyperuricaemic mouse model. The hyperuricaemic mice were injected with 2 U/kg insulin, and after 10 minutes, the cardiac tissues were isolated and obtained. Metformin increased the phosphorylation level of AMPK in mouse cardiac tissues (Figure 7C,D). The Western blot analysis results demonstrated a significant increase in the level of phospho‐IRS1 (Ser307) (Figure 7C, Figure 7F) and a significant reduction in the level of phospho‐AKT (Figure 7C, Figure 7E) and translocation of GLUT4 (Figure 7C, Figure 7G) in the hyperuricaemic mouse model, while metformin reversed these changes (Figure 7C,G). All of these results demonstrate that metformin protects against hyperuricaemia‐induced insulin resistance in cardiomyocytes via AMPK signalling pathways in vivo.

## 4 DISCUSSION

To the best of our knowledge, this is the first study to clearly illustrate that metformin, which is used worldwide as an antidiabetic medicine, protects against insulin resistance in cardiomyocytes induced by HUA and increases AMPK activation. Of course, we and others have previously shown that HUA induces insulin resistance in various cells, including cardiomyocytes, skeletal muscle cells and HepG2 cells.^6^, ^7^, ^8^, ^9^ Nevertheless, it has been unclear whether AMPK can regulate insulin resistance induced by HUA in cardiomyocytes in vitro and in vivo. Therefore, we used neonatal mouse primary cardiomyocytes and an acute hyperuricaemia mouse model, which is regarded as similar to humans with primary hyperuricaemia and can be utilized in translational research for human hyperuricaemia.

Hyperuricaemia is strongly associated with cardiovascular risk and a poor outcome in a variety of cardiovascular disease states, such as coronary artery disease, hypertension and heart failure.^14^, ^20^, ^21^, ^22^, ^23^, ^24^, ^25^ However, the underlying mechanisms to explain this association have been non‐existent. In our previous study,^6^ we explored the impact of HUA on glucose uptake and insulin resistance in primary cardiomyocytes and found that HUA induces oxidative stress, which plays a crucial role in the progression of insulin resistance in cardiomyocytes. Moreover, recent studies have confirmed insulin resistance as a powerful independent predictor of mortality and morbidity in patients with heart failure.^26^, ^27^ These studies demonstrated that therapeutically targeting impaired insulin sensitivity may potentially be favourable for patients with chronic heart failure. To determine whether metformin protects against insulin resistance induced by HUA in cardiomyocytes, we exposed primary cardiomyocytes to HUA, pre‐treated them with metformin and then quantified the uptake of glucose with 2‐NBDG, a fluorescent glucose analog, after insulin stimulation. We found that treatment with metformin may protect cardiomyocytes from HUA and inhibit insulin‐induced glucose uptake in cardiomyocytes. These results suggested that HUA resulted in insulin resistance in cardiomyocytes, but this change was weakened by pre‐treatment with metformin.

Metformin is well known to activate AMPK, which is expressed in a variety of tissues and cells, including cardiomyocytes, and plays a pivotal role in the regulation of cellular energy metabolism under stress conditions.^16^ Previous studies have shown that metformin reduces long‐term and high insulin‐induced insulin resistance in cardiomyocytes,^28^ and AMPK has been shown to protect against insulin resistance in skeletal muscle cells through restoration of GLUT4 translocation^29^ Consistent with the findings of these previous studies, we found that metformin could improve HUA‐induced insulin resistance in primary cardiomyocytes. As expected, this change was blocked by compound C, an AMPK inhibitor, indicating that AMPK activation was responsible for the suppression of insulin resistance in cardiomyocytes. In addition, using an acute hyperuricaemic mouse model, our study indicated that metformin improved the progression of insulin resistance induced by HUA, as shown by the glucose tolerance test (Figure 5A) and insulin tolerance test (Figure 5B).

It is of note that AMPK phosphorylation was increased by HUA in our present study. These findings are similar to previous studies,^30^ which showed that the level of AMPK phosphorylation was increased in cardiomyocytes under the condition of hypoxia for 15 hours. As we know, AMPK is an important regulator of cardiac energy homeostasis ^31^ and was activated by ATP depletion.^32^ We speculated that this is a defence response for self‐protection under stress/starvation in cardiomyocytes.

Interestingly, another activator of AMPK, AICAR, had almost the same effects as metformin, suggesting that activation of AMPK phosphorylation promoted the observed protective effect of insulin resistance in cardiomyocytes. In fact, AICAR has been demonstrated to protect against myocardial ischaemia‐reperfusion injury after myocardial infarction in animals and humans.^33^, ^34^ However, what procedures following AMPK pathway activation are involved in cardioprotection?

The first possibility is the metabolic effects of AMPK phosphorylation activation. Metformin has been demonstrated to activate the phosphorylation of AMPK in cardiomyocytes and mouse cardiac tissues. In fact, a recent study suggested that short‐term treatment with metformin protects against myocardial ischaemia‐reperfusion injury via the AMPK signalling pathway after myocardial infarction in mice.^11^ Therefore, AMPK has been considered to have various cardioprotective effects in animals and humans. Improvement in AMPK production by metformin may have mitigated the progression of insulin resistance induced by HUA. Both AICAR and metformin are reported to enhance glucose uptake in skeletal muscle cells and heart muscle cells.^9^, ^35^ Consistent with these reports, in the HUA plus insulin group, we found that pre‐treatment with metformin and AICAR nearly reverted glucose uptake to the level of the control group in cardiomyocytes. Therefore, the possibility exists that the AMPK‐induced uptake of glucose triggers improvement in insulin resistance, which is induced by HUA, followed by the restitution of the metabolic switch.

Another possibility is the improvement of the insulin‐activated Akt pathway, which is inhibited by HUA. A previous study demonstrated that phosphorylation of Akt regulates cell survival, growth and metabolism. Additionally, cardiomyocyte metabolism and growth are co‐ordinated by the integration of intracellular and extracellular signals.^35^ Furthermore, activation of Akt phosphorylation, a key protein kinase of insulin‐induced glucose uptake, modulates glucose uptake stimulated by insulin in cardiomyocytes.^6^ In the present study, we found that HUA could strongly inhibit Akt phosphorylation, translocation of GLUT4 and 2‐NBDG glucose uptake induced by insulin in primary cardiomyocytes. Our findings suggest that activators of AMPK, such as metformin and AICAR, could prevent HUA‐inhibited Akt phosphorylation, GLUT4 translocation and HUA‐reduced 2‐NBDG glucose uptake in cardiomyocytes. Moreover, an acute hyperuricaemia mouse model demonstrated inhibited Akt phosphorylation with insulin resistance and glucose intolerance.

The decreased Akt phosphorylation and translocation of GLUT4 in the HUA group were reversed in both the HUA plus AICAR group and the HUA plus metformin group, demonstrating that the activation of AMPK protects the insulin signalling pathway of Akt against inhibition by HUA in both the HUA plus AICAR group and the HUA plus metformin group.

## 5 CONCLUSIONS

Our findings illustrated that metformin protects against the progression of insulin resistance induced by HUA in cardiomyocytes and activates AMPK. These findings show an interaction between activation of AMPK induced by metformin and insulin resistance induced by HUA that could be important to our understanding of the potentiating effects of metformin on patients with insulin resistance and hyperuricaemia in clinical trials. Therefore, metformin may provide a new treatment strategy for hyperuricaemia‐related cardiovascular disease.

## 6 LIMITATION OF STUDY

In this study, we used an acute hyperuricaemia mouse model, but human hyperuricaemia is a chronic process. Further study with a chronic hyperuricaemia mouse model may be needed to clarify the association of metformin and insulin resistance induced by hyperuricaemia in heart tissue.

## CONFLICT OF INTEREST

The authors declare no conflict of interest. All authors read and approved the final manuscript.

## AUTHOR CONTRIBUTIONS

**Zhenyu Jiao:** Data curation (equal); Writing‐original draft (equal). **Yingqun Chen:** Data curation (equal). **Xie Yang:** Formal analysis (equal); Investigation (equal). **Yanbing Li:** Formal analysis (equal); Resources (equal); Software (equal). **Zhi Li:** Conceptualization (equal); Funding acquisition (equal); Methodology (equal); Writing‐review & editing (equal).
